# Diagnosis and treatment of ALT tumors: is Trabectedin a new therapeutic option?

**DOI:** 10.1186/s13046-017-0657-3

**Published:** 2017-12-22

**Authors:** Luca Pompili, Carlo Leonetti, Annamaria Biroccio, Erica Salvati

**Affiliations:** 1 UOSD SAFU, Regina Elena National Cancer Institute, Rome, Italy; 2 University of Tuscia, Viterbo, Italy; 3 Oncogenomic and Epigenetic Unit, Regina Elena National Cancer Institute, Via Elio Chianesi, 53 –, 00144 Rome, Italy

**Keywords:** Telomeres, Alt, Cancer therapy, G-quadruplex ligands

## Abstract

Telomeres are specialized nucleoprotein structures responsible for protecting chromosome ends in order to prevent the loss of genomic information. Telomere maintenance is required for achieving immortality by neoplastic cells. While most cancer cells rely on telomerase re-activation for linear chromosome maintenance and sustained proliferation, a significant population of cancers (10–15%) employs telomerase-independent strategies, collectively referred to as Alternative Lengthening of Telomeres (ALT). ALT mechanisms involve different types of homology-directed telomere recombination and synthesis. These processes are facilitated by loss of the ATRX or DAXX chromatin-remodeling factors and by abnormalities of the telomere nucleoprotein architecture. Although the functional consequences of telomerase and ALT up-regulation are similar in that they both prevent overall telomere shortening in tumors, these telomere maintenance mechanisms (TMMs) differ in several aspects which may account for their differential prognostic significance and response to therapy in various tumor types. Therefore, reliable methods for detecting telomerase activity and ALT are likely to become an important pre-requisite for the use of treatments targeting one or other of these mechanisms. However, the question whether ALT presence can confer sensitivity to rationally designed anti-cancer therapies is still open. Here we review the latest discoveries in terms of mechanisms of ALT activation and maintenance in human tumors, methods for ALT identification in cell lines and human tissues and biomarkers validation. Then, original results on sensitivity to rational based pre-clinical and clinical anti-tumor drugs in ALT vs hTERT positive cells will be presented.

## Background

Telomeres are conserved nucleoprotein structures localized at the ends of all linear chromosomes of eukaryotic cells preserving genomic information from loss and/or recombination [1]. The safeguard of telomeres is ensured by the shelterin complex, consisting of six telomere-specific proteins, which recognizes and assembles the telomeric DNA into a capped configuration preventing aberrant DNA damage response activation [2, 3]. Telomeric DNA, composed by specie-specific tandem repeats (TTAGGG in human), encounters progressive erosion at each cell division, leading cells into replicative senescence. This cascade of events represents an intrinsic limit to the proliferative potential of human somatic cells and is considered a tumor suppressive mechanism [4]. Physiologically, telomere erosion is countered by the telomerase enzyme [5, 6] which activity is tightly controlled during embryogenesis to establish a telomere length sufficient to ensure tissue homeostasis [7–9].

The rescue of replicative senescence is regarded as a hallmark of cancer and the majority of cancer cells overcome telomere erosion by re-activating telomerase expression through transcriptional regulation. In a limited subset of tumors, telomere length is restored by activation of alternative mechanisms (ALT) [10, 11] involving homologous recombination (HR) and homology directed telomere synthesis, preferentially occurring at lagging strand, leading to heterogeneous telomere length observed in most ALT cells [12]. In addition, ALT mechanisms have been also found in adult somatic cells of different hystotypes [13, 14], than it is speculated that ALT may be a constitutive component of telomeres that coexists already with Telomerase Activation (TA) as a back-up TMM during evolution in most eukaryotes [15].

Although cellular immortalization has been considered for years a necessary step in oncogenesis, a substantial proportion of liposarcomas [16, 17], glioblastomas [18], retinoblastomas [19], and osteosarcomas [20] have been reported as negative for both TA and ALT. Recently, the existence of highly aggressive tumors showing progressive telomere erosion, not balanced by TMMs, has been definitively demonstrated. The presence of this telomeric phenotype, referred to as ever-shorter telomers (EST), allowed cells to grow for hundreds of population doublings without effects on their malignant features [21].

## Molecular mechanisms of ALT activation

**Scheme 1:**
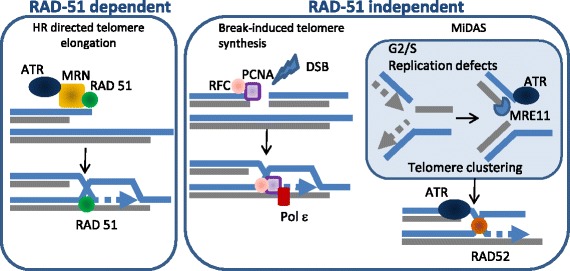
RAD51 dependent and independent ALT mechanisms

One or more alternative mechanisms for the lengthening of telomeres other than telomerase were identified spanning from yeast to human normal cells and tumors. Two types of ALT mechanisms are known in yeast [22], a Rad51-dependent mechanism, mediated by homology recombination [23], and a Rad51-independent mechanism mediated by break-induced replication (BIR) [24]. In human, ALT activity has been detected also in non neoplastic somatic cells [13] and in embryonic cells [14], and recently also in canine sarcomas [25], indicating that this mechanism is conserved among mammalians. Cancers that have a mesenchymal origin are reported to activate ALT more frequently, while epithelial cancers rely on telomerase reactivation/re-expression [26, 27]. As mesenchymal stem cells are known to express minimal or no detectable amounts of telomerase [28], and harbor less frequent TERT mutations [29], this may predispose them to depend on ALT activation more frequently. ALT are characterized by telomere associated PML bodies (called APBs) containing HR proteins, sheltering factors and heterochromatin associated proteins such as HP1 [30, 31]. Moreover, a specific phosphorylated isoform of TRF1 has been found associated with and required for APBs formation [32, 33]. Therefore, chromatin modification appears to be one of the key factors determining the choice between TA and ALT. In this regard, the presence of one or more epigenetic repressors determining the TA to ALT switch has been known from years [34]. One of the best candidate for this function has been recently identified in the alpha thalassemia/mental retardation syndrome X-linked protein (*ATRX*), death-domain associated protein (*DAXX*) and Histone 3.3 complex [35–37]. Nevertheless, the role of ATRX/DAXX and H3.3 is not completely clarified. ATRX is a chromatin remodeling protein that presents a SWI/SNF2-type ATPase/helicase and a plant homeodomain-like zinc finger. ATRX/DAXX complex localizes mainly in the nucleus and is associated with PML nuclear bodies and other subnuclear domains [38]. Functional studies shows that loss of ATRX function is necessary but not sufficient for activation of ALT [39]. Mechanicistically, it has been demonstrated that ATRX can bind and suppress R-loops at transcribed telomeres, which are more frequent in ALT [40], bind to MRN complex and contribute to the replication fork restart [41]. Recent evidence shows that ATRX knock down suppresses the NHEJ in favor of HR, contributing to the increase of replication defects and genomic instability [42–44], thus suggesting a possible mechanism of induction of ALT activity by ATRX loss of function. The Homology Recombination dependent ALT pathway in human cancer is a RAD51 mediated processes, which is similar to the yeast Type I ALT and requires the integrity of the MRN (MRE11-RAD50-NBS1) recombination complex [45, 46] (Scheme 1). In agreement with an epigenetic control in the predisposition to acquire a TA or ALT phenotype, ALT cells are characterized by overall less H3K9 and H4K20 trimethylation as well as more H3 and H4 acetylation at subtelomeric and telomeric regions. The mechanisms leading to chromatin decompaction in ALT involve the regulation of the DNMT and HDAC enzymes, the CHK1 kinase, as well as other chromatin remodelling factors reviewed in [47]. Several HR proteins were already known to be targeted by miRNA (acknowledged in [47]), although only recently, a direct role of miRNA in the TA/ALT switch has been demonstrated [48]. The different chromatin organization at subtelomeric regions lead ALT telomeres to be hyper-transcribed into long ncRNA transcripts called telomeric repeat-containing RNA (TERRA) [49]. TERRA have been implicated in the regulation of telomerase, in the formation of heterochromatin at telomeres, and in telomere stability [50]. Recently, Graf and coauthors revealed differential regulation of TERRA according to the cell cycle and to telomere length, uncovering an elegant feedback loop for telomere length maintenance [51]. Moreover, TERRA was found to bind to extra-telomeric chromatin and to influences the transcription of nearby genes; additionally, TERRA was found associated with a proteome involved in diverse processes, including chromatin remodeling and transcription [52]. TERRA R-loops forming at telomeres in yeast and human cells predispose telomeres to double-strand breaks and homology-directed repair (HDR) [53]. In some cases, HDR can drive telomere elongation and allow cells to escape senescence [54, 55]. This has led to speculation that TERRA can trigger the initiating events leading to alternative lengthening of telomeres (ALT).

Different mechanisms were proposed to explain the presence of a Rad-51 independent mechanism of ALT, similar to the yeast Type II ALT, which is mediated by break-induced replication in a POLD3-dependent [56]. Recent data show that the BLM-TOP3A-RMI (BTR) dissolvase complex is required for ALT-mediated telomere synthesis proposing that recombination intermediates formed during strand invasion initiate rapid and extensive POLD3-dependent telomere synthesis followed by dissolution, with no telomere sister-chromatid exchanges (T-SCE). This process is counteracted by the SLX4-SLX1-ERCC4 complex, which promotes resolution of the recombination intermediate, resulting in telomere exchange in the absence of telomere extension [57]. A second recently proposed mechanism, involve the telomeric mitotic DNA synthesis (MiDAS), a conservative DNA synthesis process occurring in cells defective for the G2/M checkpoint and accumulating replication stress [58] (Scheme 1). This process is coherent with the concept that ALT telomeres of cancer cells exhibit more replication defects and persistent DNA damage response. The high G/C content of telomeric sequence, harboring secondary structures, such as G-quadruplexes and R-loop formation, have been in fact regarded as triggers of replication problems at telomeres [54], which in ALT telomeres are more represented due to their increased overall length. Moreover, most ALT cells harbor frequent G2/M checkpoint mutation, overall p53 mutations [59], which are thought to be at the root of the high genomic instability of ALT cells,. This presumably allows ALT cells to enter M-phase with incomplete DNA synthesis accumulating replication stress. Mutations in the ATRX/DAXX/H3.3 genes are found associated with mutations in TP53 in cancer cells using ALT as TMM [37], and agreement with this, with this FANCJ, BRCA1 and BLM were found enriched at ALT telomeres to resolve replication stress [60].

## ALT diagnosis and prognostic value in human cancer

While TA tumors can be easily detected by TRAP assay, ALT can be deduced from the presence of different phenotypical characteristics: the absence of Telomerase activity, the presence of very long and heterogeneous telomeres, ALT-associated PML bodies (APBs), telomeric and GC-rich minisatellite instability, telomeric-sister chromatid exchange (T-SCE), and extrachromosomal telomeric DNA [41]. A summary of methodologies for ALT diagnosis in cell cultures and in tumor specimens is reported in Table 1. Extra-chromosomal telomeric DNA repeats are very abundant in ALT cells and can be linear or circular. Double stranded telomeric DNA circles (t-circles) have been found hyper-expressed in ALT cells, probably generated by telomere trimming after over-lengthening by ALT [61, 62]. Single stranded C- or G-rich circles accumulate in ALT cells as result of telomeric DNA recombination, with the C-Circles being most specific for the measurement of ALT activity. Indeed, the C-Circle Assay represents a robust and quantitative ALT assay that responds quickly to changes in ALT activity [63]. Moreover, taking advantage from polymerase amplification step, this methodology requires very small initial DNA samples and could be easily applied to biopsies or to circulating cancer cells. PML detection by immunofluorescence staining in paraffin embedded tissues, in soft tissue sarcoma samples, was shown to be a prognostic marker of poor overall survival [64]. The immunohistochemical detection of ATRX/DAXX downregulation, strongly correlates with ALT in tumor specimen of Pancreatic neuroendocrin tumors [65], in hepatic angiosarcomas [66] and leyomiosarcoma [67], suggesting that this complex could be employed in the clinical practice as a surrogate marker of ALT phenotype.

**Table 1:** Methods for ALT diagnosis in cell cultures and tumor specimens

	Cell culture	Tumor specimens
Telomerase activity	RTQ-TRAP	RTQ-TRAP
Telomere’s lenght	Southern blot Q-FISH	Q-FISH
c-circles quantification	c-circle assay	c-circle assay
TERRA detection	Northern blot RNA Fish	RNA Fish
ATRX/DAXX expression	Western blot Immunohistochemistry RTQ-PCR Microarray Deep sequencing	Immunohistochemistry RTQ-PCR Microarray Deep sequencing
APB detection	Co-IF FISH	Co-IF FISH

Regarding the clinical outcome of TMM, TA or subunit transcript expressions are generally associated with poor prognosis in breast, colorectal and lung cancer [68], but for patients with ALT, the prognosis varies among different tumor types of sarcoma and astrocytoma, where ALT shows high prevalence. In soft tissue sarcomas such as malignant fibrous histiocytomas [69], liposarcoma [70], leyomiosarcoma [67] and uterine sarcomas and carcinosarcomas [71], as well as in pancreatic neuroendocrin tumors [65], ALT is associated with decreased survival than TA. In osteosarcomas, TA or ALT presence do not differ in the clinical outcome, although the absence of TMM as expected, confer a better prognosis [20]. In contrast, presence of ALT in glioblastoma multiforme was associated with better patient outcome [18, 72], whereas in pediatric high grade gliomas ALT confers poor outcome [73] especially in association with p53 mutations [74]. It is suggested that the differential prognostic significance of ALT in gliomas may depend on the different genetic and epigenetic events responsible for activation of TMMs that is specific for the cell of origin. Moreover, emerging details on the heterogeneity of ALT molecular mechanisms can also explain the different clinical outcome.

## ALT response to treatment

**Fig. 1:**
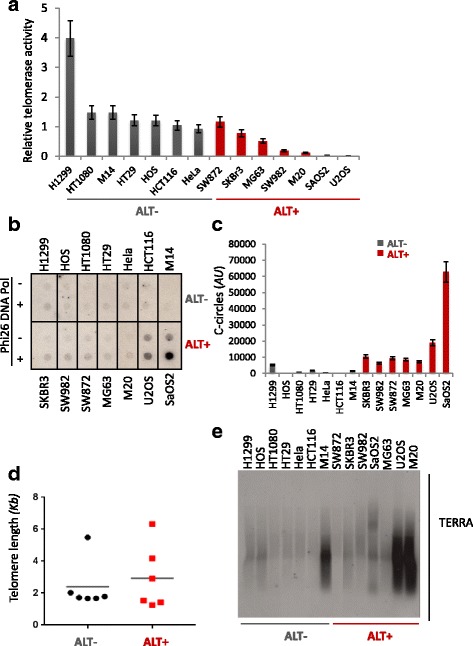
**a**. Characterization of ALT phenotypes in a panel of immortalized cell lines. Real time quantitative TRAP assay to determine the telomerase activity in the indicated cell lines. Histogram represents the fold change of telomerase activity compared to HCT116 sample. **b.** Dot blot analysis of c-circles in presence or absence of Phi26 DNA Pol enzyme in the indicated cell lines, hybridized with a ^32^P radiolabelled telo-probe. **c.** Densitometry of c-circles signals. For each cell line the background value (-Phi26 DNA Pol sample) was subtracted and reported in histograms. **d.** Quantitative analysis of the average telomere length in the indicated cell lines. Each sample underwent Southern blot analysis of the Telomere restriction fragments with a ^32^P radiolabelled telo-probe. Signals were quantified to determine the average telomere length reported by histograms. **e.** RNA samples extracted from the indicated cell lines underwent Northern blot analysis. The filter was hybridized with a ^32^P radiolabelled telo-probe for TERRA detection and the signals were acquired by Phosphoimager

One of the unanswered question regarding the presence of ALT in human cancers is whether ALT telomeres can be specifically targeted in cancer therapy. With the aim of addressing the possibility that ALT features confer different sensitivity to anti-tumor treatments, a panel of human cancer cell lines of different hystological origin were employed. H1299 (lung), HOS (osteosarcoma), HT1080 (fibrosarcoma), Hela (cervix cancer), HCT116 and HT29 (colorectal cancer), M14 (melanoma) are reported as TA positive in several papers. SKBr3 (breast cancer), SW982 (synovial sarcoma), SW872 (liposarcoma) and MG63 (osteosarcoma), although reported to express TA in different extents, show ALT activity in terms of C-circle expression (ATCC source) or PML expression [75]. SaOS2 and U2OS (oseosarcoma) are reported as ALT positive and telomerase negative by several authors and finally, M2O (melanoma) cell line was a clone obtained by over-expressing an hTERT dominant negative mutant in M14 cells, then they are telomerase negative and probably developed ALT mechanisms to sustain unlimited proliferation. The cells were first characterized in parallel for telomerase activity by a RTQ-TRAP assay (Fig. 1a), that showed that, although there is a general decrease of telomerase activity in the ALT- compared to ALT+ group, many ALT cell lines display a certain level of telomerase activity, confirming that both mechanisms coexist in many tumor cells. The presence of extra chromosomal telomeric DNA resulting from ALT activity was measured by the C-circles assay (Fig. 1 b and c), that revealed to be a robust method to discriminate the ALT positive cells. On the contrary, as shown by the median telomere length measured by the TRF assay (Fig. 1 d), not all the ALT- cell lines exhibited longer telomeres, probably depending on the initial telomere length in the tissue of origin. TERRA expression, that is known to be hyper-activated in ALT, was measured by northern blot (Fig. 1e). Also TERRA expression is not a univocal characteristic of ALT, varying among the different cell lines analyzed. Unexpectedly, among the ALT- cells, M14 displayed a significant TERRA level, which is further increased in the M20 isogenic telomerase negative cell line. Among the ALT+ group, TERRA signal is detectable in SKBr3, SW982, MG63, SaOS2, U2OS and M20 but not in SW872.

**Fig. 2:**
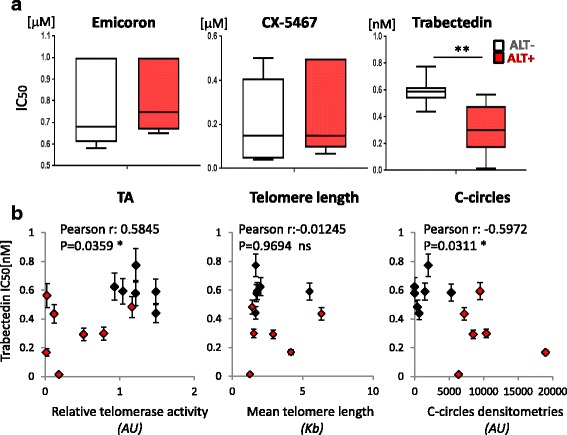
**a**. Response to G-quadruplex ligands and Trabectedin treatment in ALT and non ALT cell lines. ALT positive (SK Br3, U2OS, SW872, SW982, MG63, Saos2, M20) and negative (HCT116, H1299, HeLa HT29, HT1080, HOS, M14) cell lines were treated with Emicoron (dose range from 0,2 to 1,4 μM), CX-5461 (dose range from 0,01 to 1 μM) and Trabectedin (dose range from 0,15 to 2,4 nM) for 120 h, and then fixed and processed for crystal violet staining. Surviving fraction of cells was determined as percent of treated vs untreated absorbance values in each condition. Then the IC_50_ dose (the dose able to kill the 50% of cells compared to untreated sample) was calculated by Calcusyn software. The IC_50_ doses of compounds were compared in the ALT- and ALT+ groups of cell lines and results are shown in the box and whiskers plot (** = *P* < 0,005). **b.** Correlation between IC_50_ doses of Trabectedin and the indicated parameters (black: ALT-; red: ALT+). The “Pearson r” coefficient of correlation was calculated by the GraphPad Prism 7.03 software

The heterogeneity of ALT characteristics in cell lines of different origin support the existence of a complex variety of ALT mechanisms and pose the question whether specific ALT phenotypes can be predictive of sensitivity to treatments with specific DNA targeting drugs. As explained above, longer telomeres and the high recombination frequency, also increased by TERRA transcripition, are consistent with a higher replication stress, telomeric fragile sites, DNA-RNA hybrids (R-loops) and secondary structures (G-quadruplexes). These last are specifically targeted by an heterogeneous group of molecules with validated anti-proliferative activity, the G-quadruplex ligands. They target both telomeric and non telomeric secondary structures which are conserved in regulatory regions such as promoters, intronic regions, rDNA and untranslated 5′ and 3′ ends of mRNAs [76]. This class of compounds has been shown to target both TA and ALT tumors [77] but the different activity in ALT vs TA cells was never assayed. The mechanism of action and the telomere specific effect of this class of molecules was deeply investigated in the last years by these authors [78, 79]. G-quadruplex ligands have been shown to affect telomere capping and to perturb telomere replication, leading to an anti-proliferative effect [80, 81]. They also synergize with replication stress inducing agents like camptothecin [82] and with ionizing radiations [83]. Emicoron is a G-4 ligand with high affinity for both telomeric and non telomeric G-quadruplex with interesting anti-tumoral properties [84] evaluated in in vivo advanced models of colorectal cancer Patient Derived Xenografts [85]. CX-5461, already known as a RNA pol I inhibitor, was recently discovered to stabilize G-quadruplexes, with specific toxicity against BRCA deficiencies in cancer cells [86], as already described for other G-quadruplexes [87], including tumours resistant to PARP inhibition. Trabectedin is a tetrahydroisoquinoline alkaloid derived from the Caribbean marine tunicate, *Ecteinascidia turbinata*. Trabectedin interacts with the minor groove of DNA double helix and alkylates guanine at the N2 position, triggering a cascade of events that interferes with several transcription factors, DNA binding proteins, and DNA repair pathways, resulting in cell cycle arrest and apoptosis [88]. Trabectedin is a therapeutic option in soft tissue sarcoma [89], where ALT presence is associated with poor prognosis and has been suggested as a marker for patient stratification [90]. The effect of Emicoron, CX-5461 and Trabectedin on cell viability was measured in a crystal violet assay to calculate the IC_50_ doses of the compounds in each cell line. In the Fig. 2a the IC_50_ of ALT- and ALT+ cells are plotted, showing that ALT+ cells are significantly more sensitive to Trabectedin then ALT-, while there is no significant difference in sensitivity of the two groups to G-quadruplex stabilizers. To understand if sensitivity to trabectedin is linked to one specific ALT parameter among those analyzed, the degree of correlation between the IC_50_ doses and the TA, mean telomere lenght and c-circles expression respectively. The statistical analysis revealed that Trabectedin IC_50_ positively correlated with TA and inversely correlated with c-circles expression, while any significant correlation was found with the mean telomeres length (Fig. 2b).

## Conclusions

The requirement for cancers to utilize a TMM begs the question whether such pathways can be targeted for clinical purpose. While telomerase targeting approaches have been pursued for years leading to clinical applications, the prognostic and therapeutic significance of ALT is still debated. The emerging complexity of ALT mechanisms can explain the heterogeneous behavior of ALT tumors in terms of disease progression and response to treatment. Therefore, the deep comprehension of the molecular mechanisms at the root of ALT pathways appears to be crucial for the identification of new surrogate markers for ALT diagnosis and for the development of target specific anticancer strategies.

The results of the present work confirmed that the c-circle presence is the most sensitive and reliable method to detect ALT mechanisms also in cells expressing a certain level of TA. Moreover, we firstly described an exceptional sensitivity of ALT+ cells to trabectedin, that correlated with c-circles expression degree. Being trabectedin a drug already employed in tumor histotypes with high ALT frequency, this findings strongly suggest that ALT diagnosis in cancer patients could be predictive of treatment response and consequently help in the therapeutic choice.

## Materials and methods

### Cell cultures and treatment

The following cell lines were purchased from ATCC repository and maintained according to the purchaser’s instructions: HCT116, H1299, HeLa, SK Br3, U2OS, HT29, HT1080, SW872, SW982, HOS, MG63, Saos2. The M14 and M20 melanoma cell lines were obtained in the laboratory and maintained as described in [91]. Emicoron was synthesized and used as previously described. CX-5461 was purchased from Selleck and used according to the manufacturer’s instructions.

### Real time quantitative-telomerase repeat amplification protocol assay (RTQ-TRAP)

The SYBR Green RQ-TRAP assay was conducted as described in [92] with 0,5 μl of cell extract, (1000 cells/μl). Primer sequences were described by Kim and Wu [93]. Using the 7900HT Fast Real Time PCR System (Applied Biosystem, Waltham, MA, USA), samples were incubated for 30 min at 30 °C and amplified in 40 PCR cycles with 30 s at 95 °C and 90 s at 60 °C (two step PCR). The threshold cycle values (Ct) were determined from semi-log amplification plots (log increase in fluorescence vs cycle number) and compared with standard curves generated from serial dilutions of HCT116 cell extracts (1000, 500, 250, 50 cells). Each sample was analyzed in triplicate. Telomerase activity was expressed as fold changes of HCT116 500 cells value (Relative Telomerase Activity).

### Viability assay (crystal violet)

Cells were seeded in a 24-well plate and incubated. After 24 h, the drugs were added to the medium and allowed to grow for 96 h. Then cells were washed twice in DPBS and fixed with 4% formaldehyde for 15′ at RT. After washing with DPBS, 300 μL of crystal violet staining solution (Sigma) was added to each well, and incubated for 30′ at room temperature. Finally, the plates were rinsed twice with water, air-dryed at room temperature, and the cell pellets were dissolved in 400 μL of acetic acid. The optical density of each well in triplicate was measured at 570 nm (OD_570_) with a 96-well plate in an ELISA reader (Falcon, Corning, NY, USA). The average absorbance in each condition was used to calculate the survival expressed as percent of treated vs untreated condition. IC_50_ (the dose necessary to reduce the survival of 50%) was calculated by Calcusyn software.

### TERRA northern blot

RNA (15 μg/sample) was electrophoresed on an agarose gel and transferred onto a nylon membrane for Northern blotting analysis. Briefly, RNA (15 μg/sample) was denatured in 2.3X Denaturant (2.5 ml 40× MOPS, 2.5 ml H2O, 35 ml formaldehyde, 100 ml deionized formamide) for 15 min at 65 °C and separated by 1.2% agarose formamide gel in 1× Mops buffer at 120 V. Electrophoresis was stopped when bromophenol blue dye reached 8 cm from wells. After electrophoresis, RNA samples were transferred on nylon positively charged membrane (GE Healthcare UK Limited, Little Chalfont, UK) with 20× SSC overnight and UV cross-linked onto membrane at 125 mJ in UV crosslinker (Hoefer, Holliston, MA, USA). For RNA detection, the blot was hybridized with a ^32^P–labeled probe [T_3_AG_2_] in Church buffer (0.5 M Phosphate buffer pH 7.2, 7% SDS, 1 mM EDTA, 0.1% BSA) overnight at 55 °C. The gel was washed twice and exposed to a PhosphorImager screen and analyzed by Quantity One software (Biorad, Hercules, CA, USA).

### C-circle assay

Total DNAs were extracted using Quick C-Circle Lysis Buffer (50 mM KCl, 10 mM Tris HCl pH 8.5, 2 mM MgCl_2_, 0,5% NP40, 0.5% Tween) and treated with 0.5 μg μL^−1^ Protease (Qiagen, Hilden, Germany). Samples, 10 μl each, were combined with 10 μl 0.2 mg/ml BSA, 10% Tween, 100 mM each dATP, dGTP and dTTP, 1× Φ29 Buffer and with or without 7.5 U Φ29 DNA polymerase (NEB) and incubated at 30 °C for 4 h then 70 °C for 20 min. For quantification, the reaction products were dot-blotted onto a 2× SSC-soaked nylon positively charged membrane (GE Healthcare UK Limited, Little Chalfont, UK). DNA was UV-cross-linked onto the membrane, which was then hybridized with a ^32^P–labeled probe [T_3_AG_2_] in Church buffer (0.5 M Phosphate buffer pH 7.2, 7% SDS, 1 mM EDTA, 0.1% BSA) overnight at 55 °C. The gel was washed twice and exposed to a PhosphorImager screen and analyzed by Quantity One software (Biorad, Hercules, CA, USA).

Signals of samples without Φ29 DNA polymerase were subtracted from signal obtained from corresponding samples with Φ29 DNA polymerase to determine the c-circle expression value.

### Measurement of TRF length by southern blotting

TRF length was determined as reported in [94] with some modifications. Briefly, 15 μg sample of DNA was digested with the restriction enzymes HinfI, Alu, HPA II, Rsa I and complete cutting was confirmed by electrophoresis of DNA digests on agarose gels. Fractionated DNA fragments were electrophoresed on an agarose gel, and the gel was dried with a gel drier (Biorad, Hercules, CA, USA). The gel was denatured in 0.5 M NaOH, 1.5 M NaCl for 30 min, neutralized in 0.5 M Tris-HCl pH 7.5, 3 M NaCl for 20 min and hybridized with the same with a 32P–labeled probe [T3AG2] in Church mix (0.5 M Na2HPO4 pH 7.2, 1 mM EDTA, 7% SDS, 0.1% BSA) overnight at 55 °C. After washing twice, the gel was exposed to a PhosphorImager screen. Signals were measured using ImageQuant software and the mean telomere length was calculated as described in [94].

## Abbreviations

ALT: Alternative lengthening of telomeres
APB: ALT PML bodies
ATRX: Alpha thalassemia/mental retardation syndrome X-linked
DAXX: Death-domain associated protein
EST: Ever-shorther telomeres
HDR: Homology-directed repair
HR: Homologous recombination
MiDAS: Mitotic DNA synthesis
PML: Promielocytic leukemia
TA: Telomerase activity
TERRA: Telomeric repeat-containing RNA
TMM: Telomere maintenance mechanisms
T-SCE: Telomere sister-chromatid exchanges
